# A case report of an extremely rare association of ankylosing spondylitis with pachydermoperiostosis

**DOI:** 10.1002/ccr3.7375

**Published:** 2023-05-20

**Authors:** Faiq I. Gorial, Nabaa Ihsan Awadh, Maryam A. Hamzah

**Affiliations:** ^1^ Rheumatology Unit, Department of Internal Medicine, College of Medicine University of Baghdad Baghdad Iraq; ^2^ Rheumatology Unit, Department of Internal Medicine Baghdad Teaching Hospital Baghdad Iraq

**Keywords:** ankylosing spondylitis, clubbing, pachydermoperiostosis, sacroiliitis

## Abstract

**Key Clinical Message:**

We describe a case of a young man with features of pachydermoperiostosis and spondyloarthropathy. By describing this rarity, we aim to help build a database for future studies and construct a management plan that rheumatologists and clinicians can use.

**Abstract:**

This is the first case report in Iraq describing the combination of pachydermoperiostosis and ankylosing spondylitis. We report this interesting association in a 23‐year‐old male who presented with inflammatory back pain, coarse facial features, clubbing, signs of enthesitis, limitation of spine movement, and clinical and radiographic signs of sacroiliitis.

## 1 INTRODUCTION

The hereditary condition known as pachydermoperiostosis, also known as primary hypertrophic osteoarthropathy (PHO), which was first recognized in 1868 by Friedreich, is characterized by finger clubbing, periostosis, and skin thickening (pachydermia), along with several other clinical symptoms like hyperhydrosis, joint pain, arthritis, cutis verticis gyrata, ptosis, and hypertrophic gastritis.^1^, ^2^, ^3^, ^4^ Three forms have been described: a complete form that consists of digital clubbing, periostosis, and pachydermia; an incomplete form (no pachydermia); and a frustra form (few skeletal manifestations with prominent pachydermia).^5^ To the best of our knowledge, there have only been two documented cases in the literature that describe the combination of ankylosing spondylitis and PHO.^6^, ^7^

The first case of ankylosing spondylitis in an Iraqi patient with complete PHO is described in this article.

## 2 CASE REPORT

A 23‐year‐old man presented to the Baghdad Teaching Hospital's rheumatology consultant clinic with a 5‐year history of lower back pain that was insidious in onset, occurring mainly at rest and in the early morning, was partially relieved by activities associated with morning stiffness for about 2 h and was initially responding to prescribed non‐steroidal anti‐inflammatory medications. He reported frequent attacks of bilateral knee joint pain that was gradual in onset, with intermittent swelling associated with both legs and heel pain. There were also a few bouts of nausea and vomiting, as well as dull upper abdominal pain, bloating, and no weight loss. There had been no previous history of psoriasis, uveitis, recurrent urinary tract infections, or bloody diarrhea, unremarkable respiratory, cardiac, neurological, or family history.

**FIGURE 1:**
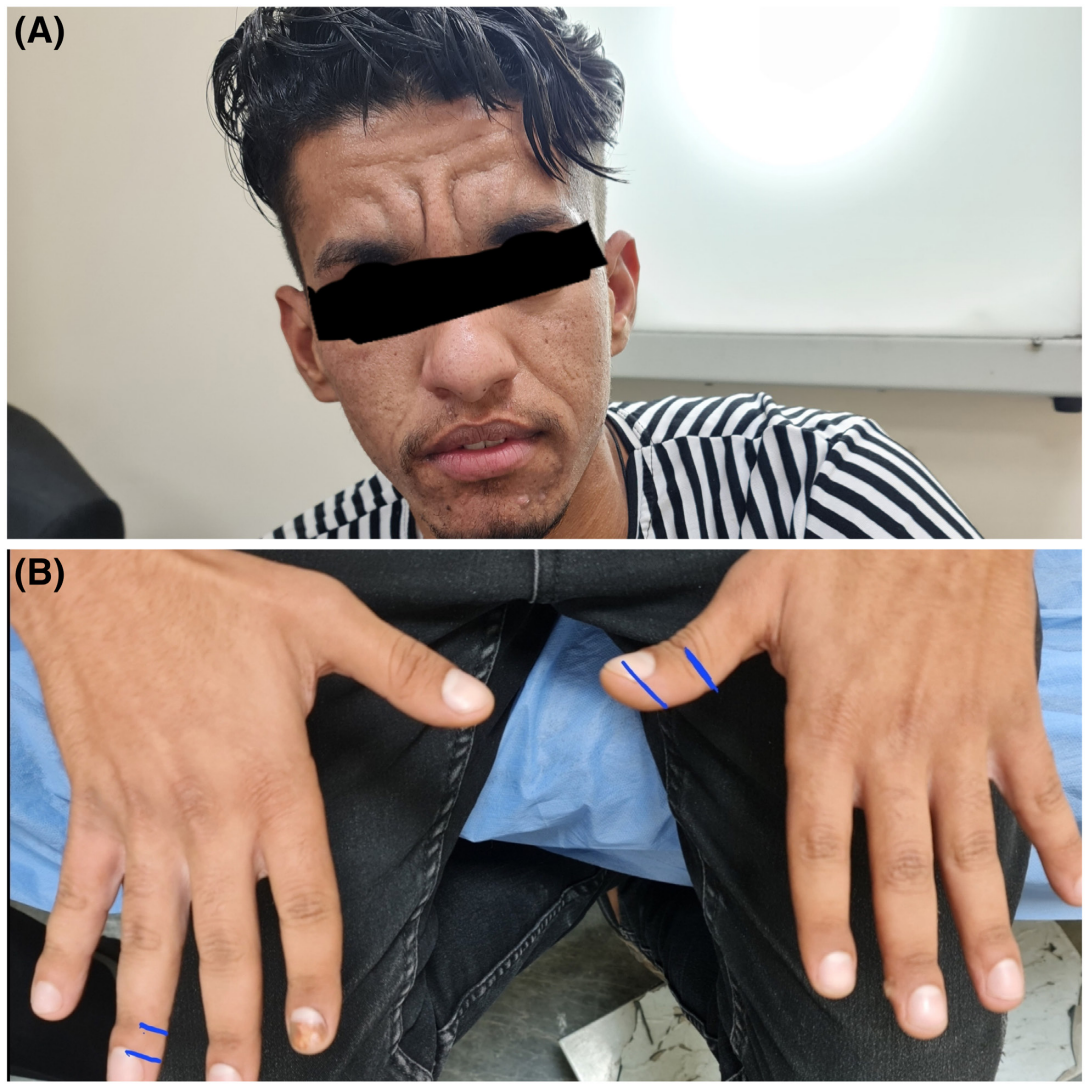
(A) An image of the patient's face reveals thickening and furrowing of the forehead skin (leonine facial features). (B) Digital clubbing.

At physical examination, the patient's vital signs and body mass index were within normal ranges. There were coarse fascial features in the form of skin thickening and prominent folds and furrows in the forehead, between the eyes, and in the nasolabial groove, indicating a leonine face, as well as an acneform rash. There was stage 3 finger clubbing,^8^ and the phalangeal depth ratio (the ratio between the depth of the distal phalynx and the depth of the DIP) was >1 (Figure 1).

The gait of the patient was normal, with preservation of the normal cervical lordosis and thoracic kyphosis but the loss of lumber lordosis. Palpation of the spine reveals tenderness in the spinous processes of the lower lumbar and sacral spine. The lateral flexion and forward flexion were restricted (modified Schober test = 17 cm). The direct sacroiliac compression test was positive, the Patric test was negative, and chest expansion was not restricted. Tenderness on palpation of both greater trochanters, the medial aspects of both knees and the heels indicated enthesitis. There were no signs of arthritis in the peripheral joints.

**FIGURE 2:**
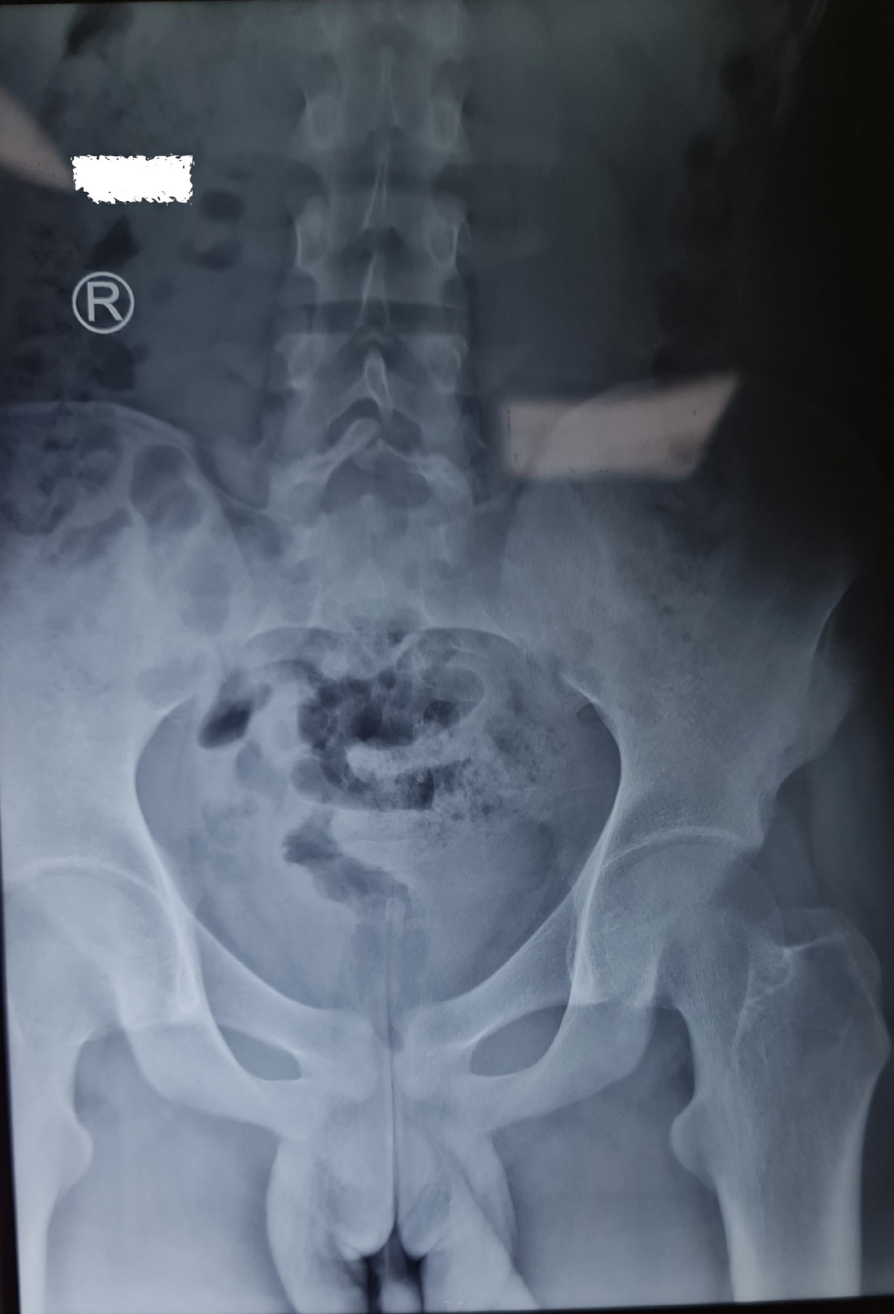
A pelvic radiograph revealed grade 3 sacroiliitis.

**FIGURE 3:**
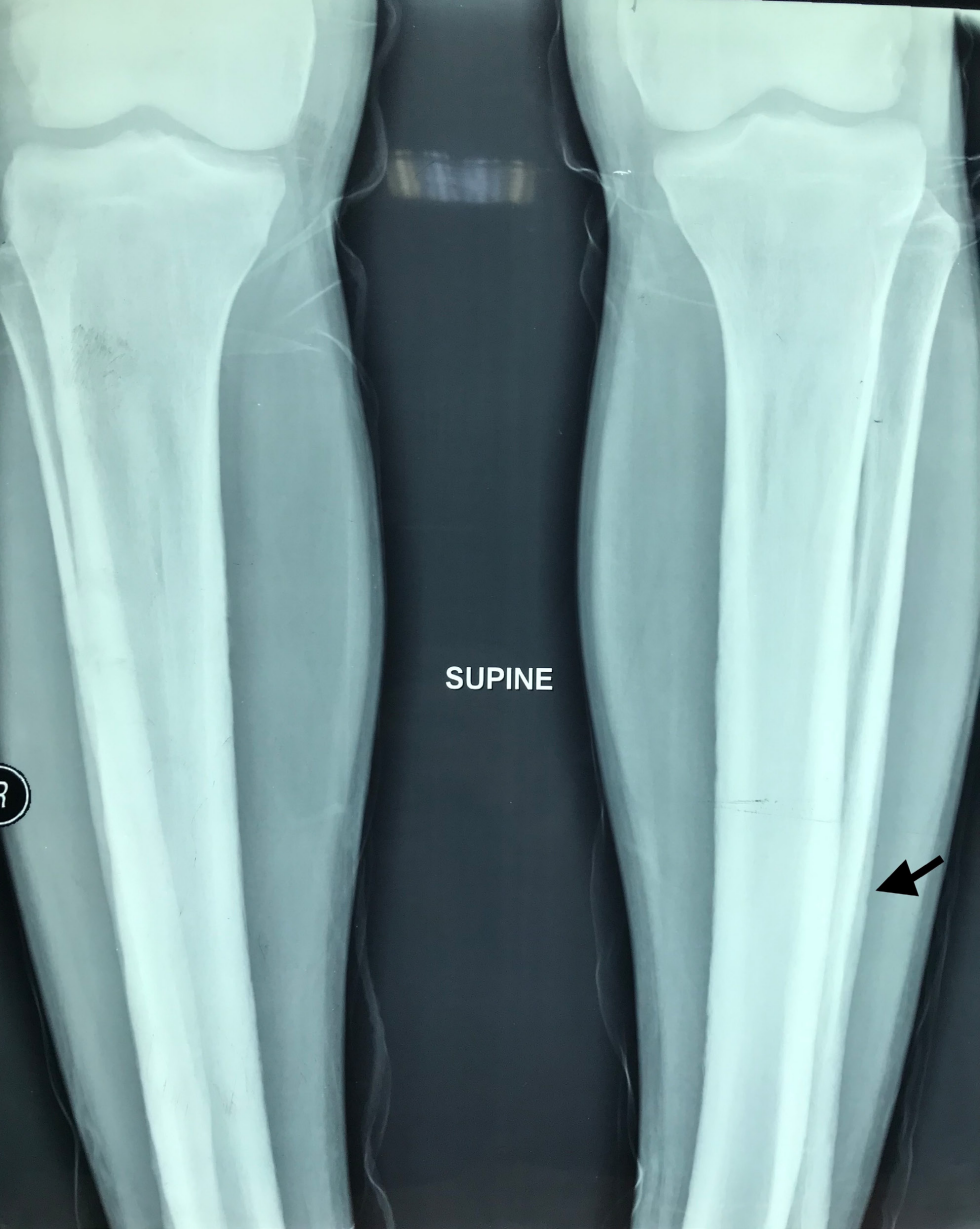
A radiograph revealed cortical thickening of the tibia and fibula, with periosteal bone deposition more apparent on the left fibula (arrow).

Laboratory investigations revealed an elevated C‐reactive protein of 45 mg/L (normal: 0–5 mg/L) and an erythrocyte sedimentation rate of 37 mm/h, while his complete blood picture parameters, renal, liver, and thyroid functions, electrolytes, and urinalysis were within normal ranges. Fecal occult blood and calprotectin were both negative. Rheumatoid factor, anti‐citrullinated peptide antibody, and anti‐nuclear antibody were negative. Mucosal congestion features are suggestive of gastritis were revealed by oesophagogastroduodenoscopy. A pelvic radiograph revealed grade 3 sacroiliitis (Figure 2). The long bones radiograph revealed cortical thickening of the tibia and fibula, with periosteal bone deposition more visible on the left fibula (Figure 3). The echocardiograph and chest CT scan were normal.

The patient was diagnosed with ankylosing spondylitis after meeting the modified New York criteria^9^ associated with pachydermoperiostosis based on clinical features, radiographic findings, and a workup to rule out paraneoplastics. He was scheduled to receive a tumor necrosis factor inhibitor along with an intravenous bisphosphonate.

## 3 DISCUSSION

Although the prevalence of pachydermoperiostosis in the general population is unknown, it accounts for about 5% of all cases of hypertrophic osteoarthropathy. It is primarily autosomal dominant in nature, with a pronounced male predominance (7–9:1) and familial aggregation occurring in 25%–38% of instances.^4^ In this case report, a 23‐year‐old male patient had back pain, progressive finger enlargement, and a gradual coarsening of facial features. The patient was diagnosed with ankylosing spondylitis associated with PHO based on the presence of chronic inflammatory back pain for more than 3 months, with sacroiliatis evident by pelvic radiography and spondyloarthropathy features (limitation of lumbar spine motion, enthesitis, and absence of rheumatoid factor). We had several differential diagnoses for his clubbing and fascial features, including acromegaly (which is characterized by larger bones of the face, skull, and limbs with jaw prognathism and high IGF‐1 with hormonal disruption), which was ruled out. It can also mimic certain types of psoriatic arthritis, such as psoriatic onychopachydermoperiostitis, but it is limited to the extremities and characterized by psoriatic nail involvement, which was not present in our patient. Thyroid acropachy was another differential diagnosis that can manifest as periostosis, clubbing, and arthralgia, closely resembling pachydermoperiostosis, but in our patient, clinical and lab evaluations (euthyroid) made it unlikely. Hypertrophic osteoarthropathy (secondary form), is caused primarily by lung neoplasia and, to a lesser extent, hepatic cirrhosis, both of which had been ruled out. Moreover, retinoids, which are linked to induce acute arthritis and sacroiliitis,^10^ were not previously used by the patient. Primary hypertrophic osteoarthropathy was the explanation for his clinical and radiographic features.

Due to the unidentified pathophysiology, pachydermoperiostosis has limited therapeutic choices. Standard medications such as non‐steroidal anti‐inflammatory medicines and colchicine are frequently used as first‐line treatments.^11^ Parenteral bisphosphonates have been shown to improve PHO‐related pain in several case studies. Although their mode of action in PHO is yet unknown, it is widely recognized that bisphosphonates have anti‐resorptive and anti‐inflammatory properties.^11^, ^12^, ^13^, ^14^

Only two occurrences of pachydermoperiostosis linked with spondyloarthritides have ever been documented,^6^, ^7^ as shown in Table 1, which could be a coincidence, but a link between these two conditions could present diagnostic challenges.

**TABLE 1:** Pachydermoperiostosis cases associated with spondyloarthritides in the literature.

Authors	Publication year	Study type	Pachydermoperiostosis form	Age (years)	Gender	Treatment
Shinjo et al.^6^	2007	Concise report	Incomplete	50	Male	Not mentioned
Zhang et al.^7^	2013	Case report	Complete	18	Male	A combination of zoledronic acid and a synovectomy

We report this case to raise awareness about this really unusual and special association.

## 4 CONCLUSION

Our case illustrates a rarely addressed or documented association between pachydermoperiostosis and ankylosing spondylitis in the medical literature. By disclosing this exceedingly unusual instance, we intend to help create a record for future studies and construct a management strategy that rheumatologists and physicians can use to better manage the condition.

## AUTHOR CONTRIBUTIONS

**Faiq I. Gorial:** Conceptualization; data curation; methodology; project administration; resources; supervision; validation; writing – review and editing. **Nabaa Ihsan Awadh:** Conceptualization; data curation; project administration; resources; validation; visualization; writing – original draft; writing – review and editing. **Maryam A. Hamzah:** Data curation; resources; software; writing – original draft.

## FUNDING INFORMATION

No source of funding has been received.

## CONFLICT OF INTEREST STATEMENT

The authors have no conflicts of interest to declare. All co‐authors have seen and agree with the contents of the manuscript.

## CONSENT

Written informed consent was obtained from the patient to publish this report in accordance with the journal's patient consent policy.

## Data Availability

Non‐applicable
